# Conservation Planning for Biodiversity and Wilderness: A Real-World Example

**DOI:** 10.1007/s00267-015-0453-9

**Published:** 2015-04-03

**Authors:** Silvia Ceauşu, Inês Gomes, Henrique Miguel Pereira

**Affiliations:** 1 German Centre for Integrative Biodiversity Research (iDiv) Halle-Jena-Leipzig, Deutscher Platz 5e, 04103 Leipzig, Germany; 2 Institute of Biology, Martin Luther University Halle-Wittenberg, Am Kirchtor 1, 06108 Halle (Saale), Germany; 3 Centro de Biologia Ambiental, Faculty of Sciences, University of Lisbon, Campo Grande, 1749-016 Lisbon, Portugal; 4 Centro Interuniversitário de História da Ciências e Tecnologia, Faculty of Sciences, University of Lisbon, Campo Grande, 1749-016 Lisbon, Portugal; 5 Departamento de Engenharia Civil e Arquitectura, Instituto Superior Técnico, Avenida Rovisco Pais, 1040-001 Lisbon, Portugal

**Keywords:** Area prioritization, Conservation management, Complementarity, Conservation planning, Protected areas, Wilderness, Zoning

## Abstract

Several of the most important conservation prioritization approaches select markedly different areas at global and regional scales. They are designed to maximize a certain biodiversity dimension such as coverage of species in the case of hotspots and complementarity, or composite properties of ecosystems in the case of wilderness. Most comparisons between approaches have ignored the multidimensionality of biodiversity. We analyze here the results of two species-based methodologies—hotspots and complementarity—and an ecosystem-based methodology—wilderness—at local scale. As zoning of protected areas can increase the effectiveness of conservation, we use the data employed for the management plan of the Peneda-Gerês National Park in Portugal. We compare the approaches against four criteria: species representativeness, wilderness coverage, coverage of important areas for megafauna, and for regulating ecosystem services. Our results suggest that species- and ecosystem-based approaches select significantly different areas at local scale. Our results also show that no approach covers well all biodiversity dimensions. Species-based approaches cover species distribution better, while the ecosystem-based approach favors wilderness, areas important for megafauna, and for ecosystem services. Management actions addressing different dimensions of biodiversity have a potential for contradictory effects, social conflict, and ecosystem services trade-offs, especially in the context of current European biodiversity policies. However, biodiversity is multidimensional, and management and zoning at local level should reflect this aspect. The consideration of both species- and ecosystem-based approaches at local scale is necessary to achieve a wider range of conservation goals.

**Electronic supplementary material:**

The online version of this article (doi:10.1007/s00267-015-0453-9) contains supplementary material, which is available to authorized users.

## Introduction

Biodiversity is facing tremendous threats from human-induced causes all over the world (Butchart et al. 2010; Pereira et al. 2010). In this context, academia, international organizations, and donors work intensely toward setting priorities in order to maximize the impact of conservation efforts (Meir et al. 2004; Halpern et al. 2006; Wilson et al. 2006). But despite the increased complexity of area prioritization methodologies and their growing implementation (Pressey and Bottrill 2008), indicators suggest little success in limiting the loss of biodiversity and ecosystem services (Butchart et al. 2010).

Designating biodiversity hotspots is one of the best known approaches. It is based at global scale on measures of species endemism and habitat loss (Myers et al. 2000), and at smaller scales on species richness and species rarity metrics (Rey Benayas and de la Montana 2003; Kati et al. 2004). The systematic conservation planning approach added complementarity into the site selection process as a measure of the contribution of a particular area to the overall unrepresented conservation targets, thus increasing the area efficiency of conservation areas (Ferrier et al. 2000; Margules and Pressey 2000). Wilderness methodologies on the other hand use continuous measures of the intensity of human encroachment in order to select the areas that have experienced the lowest impact of human presence and modern technologies (Klein et al. 2009; Watson et al. 2009). The aim is to protect those ecosystems that are closest to their natural state, have the most complete trophic networks, and therefore are still supplying specific regulating, supporting, and cultural ecosystem services (Naidoo et al. 2008; Watson et al. 2009, 2011).

When compared at bigger scales, these approaches, hotspots and complementarity on one hand and wilderness on the other, lead to different conservation priorities (Mittermeier et al. 2003; Brooks et al. 2006; Klein et al. 2009). Brooks et al. (2006) explain these differences as opposing attitudes toward vulnerability, with approaches like hotspots prioritizing areas of high vulnerability and wilderness approaches prioritizing areas of low vulnerability. However, another important conceptual difference between these approaches is the type of biodiversity dimensions that they are maximizing. While hotspots and complementarity have been designed to maximize separate ecosystem features such as species and vegetation types (Margules and Pressey 2000; Myers et al. 2000), wilderness methodologies address a composite quality of ecosystems (Aplet et al. 2000). There are few attempts to evaluate prioritization methodologies together and the focus has been mainly on species-based approaches (Kati et al. 2004; Diniz-Filho et al. 2006). When the comparisons have been more inclusive, the assessment was done unidimensionally against only one biodiversity criterion such as species richness (Klein et al. 2009; Watson et al. 2009) or ecosystem services (Naidoo et al. 2008).

In protected areas, much of the biodiversity management is done through land planning and land zoning in order to reconcile conservation actions with human use (Watts et al. 2009). Although zoning methodologies have been increasingly applied across a wide range of ecosystems (Salm and Siirila 2000; Villa et al. 2002; Linnell et al. 2005; Del Carmen et al. 2007; Geneletti and van Duren 2008; Watts et al. 2009), we lack a robust multidimensional comparison at local scale of zoning methodologies inclusive of ecosystem-based approaches. This is an important gap as zoning of established protected areas can have significant impacts on the results of conservation actions through higher resource efficiency, simplified management procedures, and higher predictability for the plans of local communities (Linnell et al. 2005).

**Fig. 1:**
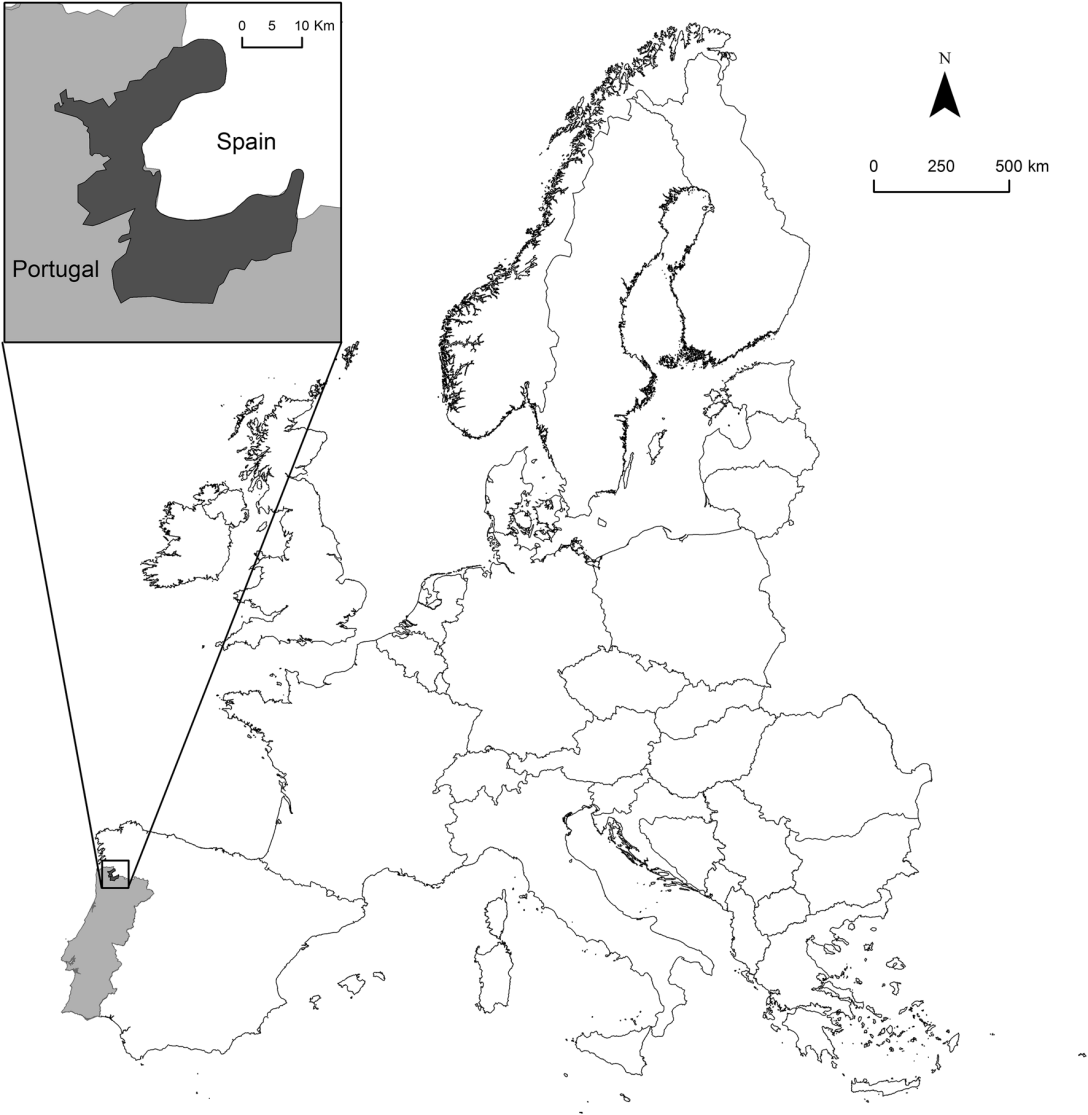
The location of Peneda-Gerês National Park in the north of Portugal

Our research addresses the following research question: is one type of prioritization approach sufficient to reach multidimensional biodiversity targets at local scale? In order to answer this question, we approach two related problems: how different are the areas prioritized by species- and ecosystem-based approaches; and which prioritization approach maximizes each of the biodiversity targets considered. For this purpose, we map and compare zoning methodologies across multiple dimensions of biodiversity at local level in the Peneda-Gerês National Park (PNPG) in Northern Portugal (Fig. 1). We analyze the prioritization methodologies according to four criteria: total bird, reptile and amphibian species representativeness; coverage of wilderness as an indicator of naturally evolving ecosystems; coverage of the important areas for megafauna; and three regulating ecosystem services. Finally, we discuss the management implications, the advantages and the drawbacks of each prioritization methodology. While there are studies using complementarity and prioritization algorithms for wilderness and ecosystem services at larger scales (Chan et al. 2006; Klein et al. 2009; Moilanen et al. 2011), we chose to use wilderness as a separate prioritizing score and ecosystem services only as a comparison criterion in order to emphasize how zoning for different biodiversity dimensions leads to different solutions.

## Methodology

### Study Area and Datasets

The area of this study is the Peneda-Gerês National Park (PNPG) in northern Portugal (longitude 8°25′W and latitude 41°41′N), the only protected area with national park status in the country (Fig. 1). PNPG was initially established as a protected area in 1971 and it is included also in the Natura 2000 network (European Council 1979, 1992).

PNPG occupies a territory of approximately 700 km^2^. The present human population living within the PNPG is approximately 8800 inhabitants (Instituto Nacional de Estatística 2011). Low-intensity agriculture and extensive grazing have been economically unproductive and the region is currently undergoing significant changes due to farmland abandonment. A significant area of the park has been classified as High Nature Value farmland by the European Union (European Environment Agency 2004). Habitat composition contains Atlantic and Mediterranean habitat types.

For the hotspots and the complementarity approaches, the territory of PNPG was divided in a grid of UTM quadrats of 2 km × 2 km, the highest resolution common to all species data. We used presence-absence data covering 13 species of amphibians, 20 species of reptiles, and 144 species of birds. Out of the total of 233 quadrats included in the analysis, information was missing for 13, 11, and 16 quadrats for birds, reptiles, and amphibians, respectively. The species data are atlas distribution data collected at the level of PNPG and published in Pimenta and Santarém (1996) for birds, and in Soares et al. (2005) for herpetofauna. The data represent recorded presences through multi-year monitoring of the territory of the park based on several methodologies (visual encounter surveys, calls surveys, search of potential shelters). The data also include ad hoc observations by the authors and the staff of PNPG. The data do not include abundance records.

For wilderness mapping, we rasterized the territory of the PNPG and the adjacent area in a grid with a pixel resolution of 10 m^2^. We based the analysis (see below) on infrastructure data extracted from maps of the Portuguese Geographical Institute of the Army (Instituto Geográfico do Exército 1997).

We defined megafauna as the species in PNPG with the largest body mass for which we had data (PNPG-ICN 2008). As such, we used point data for locations of dens of wolf *Canis lupus* (Linnaeus, 1758), and past and present nesting sites for the eagle-owl *Bubo bubo* (Linnaeus, 1758), and golden eagle *Aquila chrysaetos* (Linnaeus, 1758). These data are based on annual monitoring of the wolf population and annual surveys of the nests of the birds of prey (PNPG-ICN 2008). We also used polygon data for important areas for wild goat *Capra pyrenaica* (Schinz, 1838), which were defined based on habitat characteristics (Moço et al. 2006). We created a buffer of 1 km around the point locations and we merged these buffer areas with those important for the wild goat. We chose this size of the buffer based on the literature on the effects of human disturbances on wolves and birds of prey (Thiel et al. 1998; Martínez et al. 2003; Penteriani et al. 2005; Ruddock and Whitfield 2007; Iliopoulos et al. 2014).

We used a digital elevation model (DEM) to define areas important for landslide protection (Earth Remote Sensing Data Analysis Center 2011) by prioritizing terrains with slopes steeper than 30°. We merged these areas with spring protection areas and groundwater recharge areas, which were calculated by the administration of PNPG based on the methodology described in Brilha (2005). The calculation was done based on land use, slope and elevation, hydrology of the area, and data collected from 130 locations across the park (PNPG-ICN 2008). These data refer to the supply of ecosystem services. The local population utilizes these ecosystem services through the use of local water and soil resources but the available data do not make it possible to estimate the spatial variation in the use of ecosystem services.

We used the ArcGIS 10 software package (Esri, CA, USA) for mapping and spatial analysis. We used MARXAN software (Ball et al. 2009) for applying the complementarity prioritization approach (Ardron et al. 2008). Statistical analyses were carried out in the R software package (R Development Core Team 2011).

### Species-Based Approach: Hotspots

We calculated the number of species present and an average rarity and vulnerability for each grid cell. The rarity value of each species was the inverse of the number of cells in which the species was present. We assigned vulnerability scores to species on a scale from 0 to 10 according to the national red list (Cabral et al. 2005). We gave the least concern species the score 0 and to the critically endangered the maximum score of 10. We assigned scores to the next two threat categories at an equal distance of two units: 8—threatened, 6—vulnerable. Both near threatened and data-deficient categories contain species which cannot be assigned to a threatened category but which can also not be considered of least concern due to lack of data or due to impeding future threat. Thus, we combined these species into one mixed bag category, and we gave it the middle vulnerability score between least concern and vulnerable—3. We increased the difference in units compared to the threatened categories but, in the same time, we gave it a higher vulnerability score than the least concern category because it contains species that might be threatened presently or in the future. We assigned the value corresponding to the data-deficient class to the species for which information was not available. The choice of the scoring methodology does not have a strong impact on the ranking of the grid cells based on the hotspots methodology (Online resource 1).

We normalized the richness, average rarity, and average vulnerability into the [0,1] interval according to the formula:

1 $$x_{\text{n}} = \frac{{x - x_{\hbox{min} } }}{{x_{\hbox{max} } - x_{\hbox{min} } }},$$

where *x*_n_ is the normalized value, *x* is the initial value, and *x*_min_ and *x*_max_ are the minimum and the maximum values across all species.

We prioritized the grid cells using AI = SR_n_ + *R*_n_ + *V*_n_, where AI is the aggregated index according to which we define biodiversity hotspots, and SR_*n*_, *R*_*n*_, and *V*_*n*_ are the normalized values for species richness, rarity, and vulnerability, respectively, for each grid cell. We decided to give them equal weight in our calculation because species richness, rarity, and vulnerability are all frequently used in conservation prioritization, many times jointly (Williams et al. 1996; Lawler et al. 2003; Brooks et al. 2006), but they often prioritize different areas without a consensus on which metric is better at capturing conservation value (Lennon et al. 2004; Orme et al. 2005).

### Species-Based Approach: Complementarity

For the complementarity analysis, we simplified the vulnerability scoring used for the hotspots methodology. We classified as vulnerable all species which were not included in the least concern category of the national red list (56 out of 177 species). After several test runs, we considered a coverage of 50 % of the total number of occurrences of each vulnerable species and 10 % of the occurrences of each non-vulnerable species. We chose these percentages because they were the highest values for which all representation targets were fulfilled while allowing enough variation in the different sets of selected areas (Ball et al. 2009). We set the target representation at 100 % for the species present in only one planning unit and we considered the costs of all planning units equal to unity. We performed 2000 runs of the MARXAN software and we used only the results meeting all the conservation targets. We then used the frequency of selection of each cell, also known as summed irreplaceability (Pryce et al. 2006; Ardron et al. 2008), as the prioritizing score.

### Species-Based Approach: Wilderness

We used five infrastructure elements: the primary and secondary road networks, the human settlements, the power grid, and the hydroelectric dams. We chose these elements based on the local context of the park and on the literature (Fritz et al. 2000). Other elements used in the wilderness mapping literature, especially at larger scales, include railroads, human population density, biophysical naturalness based on expert opinions, and size of ecologically intact regions (Sanderson et al. 2002; Mittermeier et al. 2003; Woolmer et al. 2008). We expect such metrics to be highly correlated to the wilderness value calculated based on our selected infrastructures (e.g. human population density) or to be irrelevant for the scale of our study area (e.g. size of ecologically intact regions). We included in the analysis both the infrastructure inside the territory of the park, and the infrastructure found in the proximity of the park and which was likely to have an impact inside PNPG. As such, the external infrastructures were located in an air distance radius around the park of approximately 20 km in the case of the primary road network, and approximately 10 km in the case of the secondary road network, the power grid and the human settlements. We chose to consider infrastructures at these radiuses outside the park in order to account for both biodiversity effects and the human access and visual impact dimensions of wilderness (Fritz and Carver 1998; Cinzano et al. 2000; Carver et al. 2012).

We calculated the distance from each pixel to the nearest infrastructure of each type. We normalized the values into the interval [0,1] according to the formula:

2 $$d_{\text{n}} = 1 - \frac{1}{1 + \alpha \,*\,d},$$

where *d*_n_ is the normalized value, *d* is the distance to the closest infrastructure element of the considered type, and *α* is a scaling constant equal to 0.001. We used this value of the scaling constant in order to describe the nonlinear relationship between human infrastructures and its impacts on biodiversity (Thiel et al. 1998; De Molenaar et al. 2006; Ruddock and Whitfield 2007) and on the perception of wilderness (Cinzano et al. 2000; Kuechly et al. 2012). These impacts are strong and rapidly decreasing in the first hundreds of meters or the first kilometers, depending on the type of infrastructure. Our formula leads to a rapid decrease of human impact in the 2 km adjacent to human infrastructures and the impact reaches an asymptote beyond this distance.

We calculated the wilderness index according to the formula:

3 $$W = \sum\limits_{i} {\beta_{i} d_{i} },$$

where *W* is the wilderness score in any pixel of the map, *d* is the distance from that pixel to the closest infrastructure element of type *i*, and *β*_*i*_ is the weight assigned to infrastructure of type *i*. We assigned the weights for each infrastructure based on the assessment of the technical staff of PNPG and the impacts documented in the literature (Fritz et al. 2000; Carver et al. 2002). Thus, primary roads and human settlements had *β*_*i*_ = 1, and secondary roads, power lines, and hydroelectric dams had *β*_*i*_ = 0.25.

### Comparison of the Prioritization Approaches

The comparison of the three prioritization approaches includes the spatial congruence between the three approaches and the coverage of four biodiversity dimensions: species representativeness, wilderness coverage, coverage of important areas for megafauna, and ecosystem services. We calculated the spatial congruence between the three approaches for three levels of high-priority areas for conservation: 10, 20, and 30 % of the PNPG territory. Due to the lower resolution of the data used for the hotspots and complementarity approaches, the percentage cut-offs for the highest priority areas for these approaches have a variation from the high-priority targets of ±2 % of the total area.

We calculated Spearman’s rank correlations between the prioritizing score of each approach, species richness, rarity, and vulnerability. We averaged the wilderness scores overlapping each of the 233 grid cells and used it to calculate the correlations.

**Table 1:** Spearman’s rank correlation coefficients (*ρ* values) between the values of the prioritization parameters for the three approaches, species richness, species rarity, and species vulnerability

Parameter	Complementarity	Hotspots index	Wilderness score	Species richness	Species rarity	Species vulnerability
Complementarity		0.790***	−0.194**	0.643***	0.703***	0.415***
Hotspots index	–		−0.130*	0.628***	0.829***	0.685***
Wilderness score	–	–		−0.432***	−0.239***	0.299***
Species richness	–	–	–		0.492***	0.003
Species rarity	–	–	–	–		0.482***
Species vulnerability	–	–	–	–	–	

* *P* < 0.05; ** *P* < 0.005; *** *P* < 0.0005

We assessed the efficiency of species- and wilderness-based approaches by calculating the average percentage of each biodiversity dimension (BD) being protected per percentage unit of prioritized area. We calculated BD according to the formula:

$${\text{BD}}\,(\% ) = \frac{1}{\varLambda}\frac{{\text{BD}}_{\varLambda}}{\text{BD}}_{\hbox{max} }$$

where *Λ* is the percentage of area being prioritized; BD_Λ_ is the value of the biodiversity dimension covered by the prioritized area; and BD_max_ is the maximum value for the respective biodiversity dimension, either number of species, total wilderness value, or total important area for ecosystem services, and megafauna. We assigned *Λ* two percentage values: approximately 28 %—the minimum complementarity prioritized area that covers all the species in our list; and approximately 44 %—the minimum hotspots prioritized area that covers all the species. The percentages are approximations because of the different spatial units used for each approach but the difference between the sizes of the prioritized areas is never larger than 1 % of the total area of PNPG. Values are rounded up to two decimal places.

In order to calculate the cumulative representativeness of species, wilderness, and important areas for megafauna and ecosystem services, we converted the maps of the hotspots and complementarity approaches to rasters with a pixel resolution equal to the resolution of the wilderness map. We ranked all the points of the three prioritization maps into a *K* number of ranks of equal area, from the highest to the lowest values of the respective prioritizing score, with rank 1 representing the highest values and rank *K* representing the lowest values. Due to the high clustering of summed irreplaceability values, the value of *K* was 19 for the complementarity approach, and 25 for the hotspots and wilderness approaches. We derived the set of points belonging to each rank *K* for each map as {(*x*_*K*_^1^, *y*_*K*_^1^), (*x*_*K*_^2^, *y*_*K*_^2^), *…*, (*x*_*K*_^*n*^, *y*_*K*_^*n*^)} where *x*, *y* were the spatial coordinates of each of the *n* points of rank *K*.

We classified as rare those species that were present in less than 25 % of the total number of cells. We calculated the cumulative number of total, rare, and vulnerable species by intersecting the ranks of each prioritization map with the species data. We then counted the number of unique species covered by each rank. In the case of the hotspots and complementarity maps, the points corresponding to different ranks overlapped with the grid cells of the species data. In the case of the wilderness map, we considered a species covered by a certain rank when the points of the respective rank intersected at any rate the grid cells in which that species was present.

We calculated the coverage of the areas important for megafauna and ecosystem services by intersecting the rank points of each prioritization map with the total amount of important areas for megafauna and ecosystem services, respectively. We calculated the coverage of megafauna and ecosystem services areas for each rank *K*, weighted by the number of megafauna species and ecosystem services, respectively, present in overlapping areas.

We measured the wilderness coverage of the three approaches by intersecting the rank points of the prioritization maps with the wilderness score map. We extracted the wilderness value for each point of each rank. We then calculated the total wilderness covered by each rank according to the formula:

4 $$W_{K} = \mathop \sum \limits_{i = 1}^{{n_{K} }} W(x_{K}^{i} ,y_{K}^{i} ),$$

where *W*_*K*_ is the total wilderness score covered by rank *K* and *W*(*x*_*K*_^*i*^, *y*_*K*_^*i*^ *)* is the wilderness value corresponding to the point *i* of the *n*_*k*_ number of points corresponding to rank *K*.

## Results

**Fig. 2:**
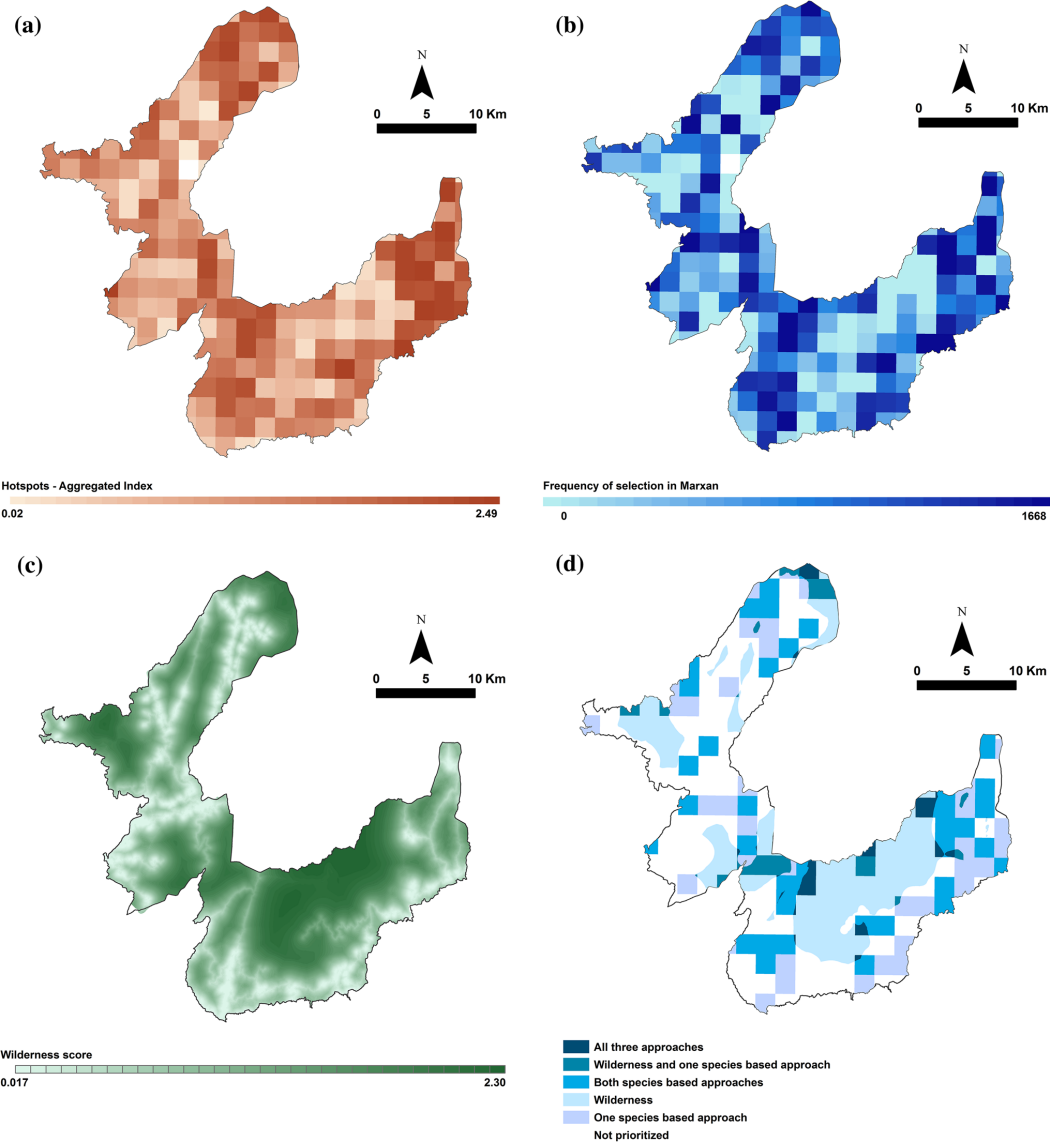
The prioritization of the territory of Peneda-Gerês National Park (PNPG) according to **a** the hotspots approach; **b** the complementarity approach; **c** the wilderness approach. **d** The spatial congruence between the three approaches at 30 % prioritized area

The species richness for each 2 km × 2 km cell ranges between one and 107 with an average of 40.7 species (standard deviation = 18.52). The 177 species have between one and 212 occurrences with an average of 53.5 occurrences. The eastern and northern parts of PNPG have a bigger number of cells spatially clustered into hotspots of species richness, rarity, and vulnerability (Fig. 2a). Between the two larger areas there is a mosaic of cells with both high and low values.

For the complementarity approach, out of the 2000 runs of the MARXAN selection, 1668 runs achieved all the conservation targets. Of these, 20 planning units were always selected and 55 cells were never selected. The complementarity values are very similar to the hotspots but the highest values are limited to a lower number of cells (Fig. 2b). The central areas of PNPG seem to increase in importance in the complementarity approach compared with the hotspots.

Highest values of wilderness are recorded in a large patch in the central part of the park, at the border of the park (Fig. 2c). The northern and western areas also show high wilderness values but confined to smaller patches. Low wilderness areas border the southern and eastern edges of PNPG. The low wilderness values in the northern and central part of the park follow the road network and human settlements location. The wilderness variation across the map is smoother than in the case of hotspots and complementarity due to the continuous values of the wilderness range.

The prioritizing scores of the hotspots and complementarity approaches are highly positively correlated (Table 1). They both show a weak but significant negative correlation with the wilderness score. There is also a high correlation between the prioritizing scores of both the hotspots and complementarity approaches, and all species indices. Wilderness score shows a relatively weak positive correlation with species vulnerability and negative correlations with species richness and rarity (Table 1). There is no correlation between species richness and vulnerability on the territory of PNPG but there is a positive correlation between species richness and rarity.

Considering the three levels of high-priority areas, only the 20 % and the 30 % prioritization levels allow for an overlap between species-based and wilderness approaches (Table 2). Even in these cases, the overlap is limited to a few percentages of the area of the park (Fig. 2d). The area prioritized commonly by hotspots and complementarity is relatively high at all three percentage levels (Table 2).

**Table 2:** Overlap between the prioritization approaches at three levels of designated high-priority areas: 10, 20, and 30 % of the total area of Peneda-Gerês National Park (PNPG)

Approaches	10 % prioritized area (%)	20 % prioritized area (%)	30 % prioritized area (%)
Wilderness + hotspots + complementarity	0	0.7	2.31
Wilderness + hotspots	0	1.52	2.42
Wilderness + complementarity	0	0	1.09
Hotspots + complementarity	6.1	12.94	17.89
Covered by at least one approach	25.17	46.04	63.29

The results are given as percentage of the total area of the park

**Fig. 3:**
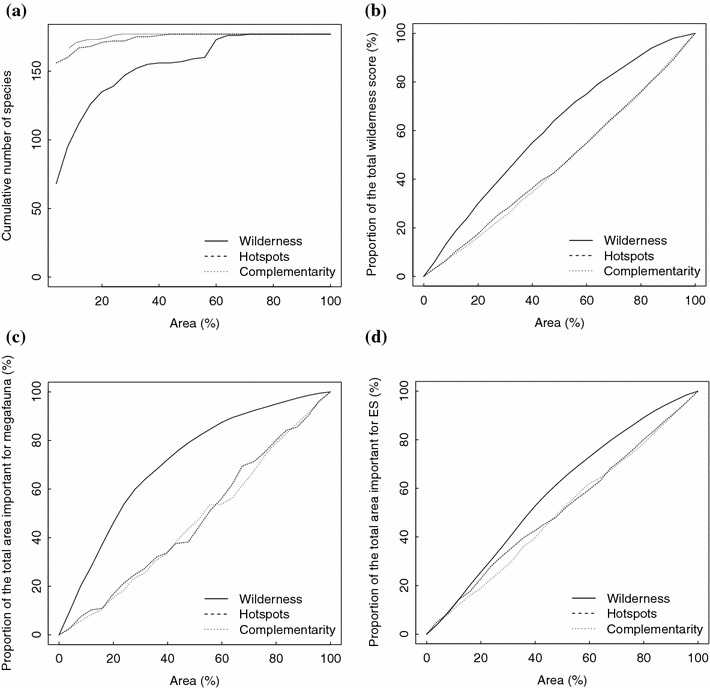
Cumulative representativeness of biodiversity criteria plotted against the percentage of prioritized PNPG area according to the species-based and wilderness approaches for **a** all species considered; **b** wilderness; **c** areas important for megafauna; **d** areas important for ecosystem services (ES)

We then assessed each approach against four criteria: species representativeness, wilderness coverage, important areas for megafauna, and ecosystem services. The species-based approaches cover all species in the smallest area (Fig. 3a, Online resource 2), while the wilderness approach covers wilderness, ecosystem services, and megafauna more efficiently (Fig. 3b–d). Complementarity is the most efficient for species protection, covering a higher number of species per percentage unit of prioritized area (Table 3). The best performance of the wilderness approach relative to the species-based approaches is the coverage of the important areas for megafauna (Table 3).

**Table 3:** Average percentages of biodiversity criteria (BD) being protected per percentage unit of prioritized area through the three approaches

Approaches	Total species (%)		Vulnerable species (%)		Rare species (%)		Wilderness (%)		Ecosystem services (%)		Megafauna (%)	
Size of prioritized area:	I	II	I	II	I	II	I	II	I	II	I	II
Complementarity	3.70	–	3.70	–	3.70	–	0.82	0.88	0.93	1.02	0.82	0.88
Hotspots	3.46	2.27	3.43	2.27	3.43	2.27	0.93	0.91	1.14	1.05	0.89	0.88
Wilderness	2.96	2	2.86	2.02	2.65	1.86	1.43	1.34	1.29	1.30	2.14	1.73

Values are calculated for two percentages of prioritized area: I—28 % and II—44 %

## Discussion

Our research compares species-based and ecosystem-based prioritization approaches used in zoning the Peneda-Gerês National Park in Northern Portugal. PNPG was initially established for the protection of wilderness (Pinto and Partidário 2012). Now it is also a Natura 2000 site, listed under both the Habitats and the Birds Directive (European Council 1979, 1992). As the national and European trend turned from wilderness to a more species-oriented approach, the subsequent management plans favored species richness and cultural landscapes (Pinto and Partidário 2012). The area selection of the European network of protected areas is debated but it has been shown to cover a significant number of threatened taxa (Araújo 1999; Araújo et al. 2007; Donald et al. 2007), while low human impact areas are inconsistently represented (Martin et al. 2008; Selva et al. 2011).

Our zoning results show the two species-based approaches prioritizing similar areas, while the ecosystem-based approach offers significantly different results. The patterns in our study area of 700 km^2^ concur with the results at global level described by Brooks et al. (2006). Moreover, the negative correlation between wilderness and species richness suggests a positive correlation between human density and species richness at the scale of our study. Other studies also find a spatial concurrence between high species richness and high human densities. In sub-Saharan Africa, species richness of mammals, birds, snakes, and amphibians is positively correlated with human population density (Balmford et al. 2001). The same is true in Europe for plant, mammal, reptile, and amphibian species richness (Araújo 2003), and for bird species richness in South Africa (Chown et al. 2003). Although there are wilderness areas which exhibit high species richness (Mittermeier et al. 2003), these do not represent most cases.

Although in some cases human management can lead to an increase of species richness (Rey Benayas et al. 2007), the generality of this pattern rather suggests that the drivers of high species richness, such as the level of primary productivity, are the same as the drivers of high human densities (Chown et al. 2003). However, the dominant view of current biodiversity policies is that European species richness is dependent on traditional agriculture (Halada et al. 2011). Therefore low-intensity agricultural practices are currently supported at European level through subsidy schemes aimed at High Nature Value farmland (European Commission 2005). But the current management actions for the maintenance of species diversity offer no guaranties as species occurrences are the complex result of a multitude of factors (Chown et al. 2003; Guisan and Thuiller 2005).

Large body mass species in particular face strong competition from humans in terms of resources (Barnosky 2008) and space (Ceballos and Ehrlich 2002) which suggests that megafauna has often better chances of survival away from human presence. For example, in a recent study, Schuette et al. (2013) find that apex predators avoid human presence through spatial and temporal niche partitioning in an area occupied by semi-nomadic human populations, while in Greece, Iliopoulos et al. (2014) showed wolves consistently avoid roads and human presence. In PNPG, wilderness areas are the preferred territory of several megafauna populations (Fig. 3c). These species play important roles in modulating trophic networks, community composition, and ecosystem properties (Duffy 2003; Schmitz 2006; Ritchie and Johnson 2009); therefore, their conservation is particularly important for ecologic processes.

The relation between species diversity and ecosystem services is complex. In the Californian Central Coast ecoregion, there are few and weak positive correlations between ecosystem services and high species diversity areas (Chan et al. 2006). At the global scale, wilderness coincides with areas important for carbon storage and sequestration, whereas hotspots better support water provision and the grassland production of livestock (Naidoo et al. 2008). At the scale of our study, the wilderness conservation approach selects a larger area important for the three regulating ecosystem services than species-based approaches (Fig. 3d), while species-directed conservation actions currently support the maintenance of low-intensity farmland (European Environment Agency 2004). Wilderness-favoring management could allow self-sustaining ecosystems and complex food webs to expand and increase resilience of ecosystems (Walker 2002) but it would lead to a decrease in provisioning ecosystem services by limiting human farming activities in the area. Such trade-offs between provisioning and regulating services have also been pointed out in the literature (Naidoo et al. 2008; Maes et al. 2012).

The drawbacks of species-based approaches are mainly related to the data used for prioritization, while the drawbacks of ecosystem-based approaches are related to their potential for social conflict. For example in our case, although the species data are the highest quality available for the zoning of PNPG, there are indications of undersampling as we have grid cells listing only one species occurrence in an area of 2 km × 2 km. In species-based approaches, there is also a strong bias toward more speciose or more charismatic taxonomic groups (Andelman 2000; Rodrigues and Brooks 2007). Such biases are common in the data available at the scale of conservation actions and the topic is hotly debated in the literature because it impacts decision making (Andelman 2000; Hess et al. 2006; Cabeza et al. 2007; Rodrigues and Brooks 2007; Roth and Weber 2008) and the consequences are still poorly understood (Gaston and Rodrigues 2003). Moreover, there are few cases in which species data were used for real-world zoning or designating of protected areas (but see Howard et al. 1997), area zoning being often done opportunistically (Hull et al. 2011). Wilderness on the other hand has the lowest requirements and uncertainty in terms of data among the tested approaches in our study. We used spatial data on infrastructures and human settlements, which are usually readily available from government agencies or geographical institutes. From an implementation point of view, ecosystem-based approaches are clearer than species-based approaches in prescribing measures for the protection of wilderness such as reducing human activities and infrastructure development in priority areas (Fritz et al. 2000). However, wilderness management actions have a high potential for social conflict, even in areas with dwindling farming populations (Navarro and Pereira 2012).

Our research shows that species- and ecosystem-based approaches prioritize different areas that maximize different biodiversity targets. However, we do not consider them as competing in conservation. As biodiversity encompasses all levels of complexity (Secretariat of the Convention on Biological Diversity 2001), conservation should address all biodiversity dimensions (Kareiva and Marvier 2003; Lee and Jetz 2008). Serious consideration must be given to the effects of possibly conflicting management actions at local scale but we consider that in areas of both high wilderness and high species richness, differentiated conservation targeting and zoning is necessary for addressing all dimensions of biodiversity.

Conservation is context dependent (Gillson et al. 2011) and contexts are extremely different across the globe. However, we are confident that prioritizing for species or ecosystem properties targets will yield similar results across the world as many mechanisms driving biodiversity and ecosystem services are common. We disagree that the goals of species conservation and wilderness should be kept distinct (but see Sarkar 1999). Wilderness areas show consistently to be important for several ecosystem services (Naidoo et al. 2008) and they contain the biological communities closest to their unaltered pre-human state (Bryant et al. 1997). Although many times these approaches are presented as mutually exclusive, we consider that they target different dimensions of biodiversity conservation. A serious consideration of species-based alongside ecosystem-based approaches in conservation management would achieve more goals than a single-minded direction, and can have important benefits for the long-term preservation of biodiversity and ecosystem services.

## Electronic supplementary material

Below is the link to the electronic supplementary material.

Supplementary material 1 (DOCX 74 kb)

Supplementary material 2 (DOCX 113 kb)
