# Supplementary material for: Conservation Planning for Biodiversity and Wilderness: A Real-World Example
Source: Environ Manage. 2015 Apr 3;55(5):1168–80. doi: 10.1007/s00267-015-0453-9 (PMC4392121; doi:10.1007/s00267-015-0453-9)
Supplement: Supplementary file 1 — Supplementary material 1 (DOCX 74 kb) [file 267_2015_453_MOESM1_ESM.docx]

Supplementary material 1

Our assigned vulnerability score is the following: 10 – critically endangered, 8 – threatened, 6 – vulnerable, 3 – data deficient and near threatened, and 0 – least concern. We assessed the sensitivity of our results to our choice of the vulnerability scoring system by defining 4 alternative scorings:

1. – no vulnerability score;
2. – all species, except the least concern ones, receive a vulnerability score equal to 1;
3. – the vulnerability scale goes from 0 to 4 with an increment of 1 for each threat level: 4 – critically endangered, 3 – threatened, 2 – vulnerable, 1 – data deficient and near threatened, and 0 – least concern;
4. – the vulnerability scale goes from 0 to 8 with an increment of 2 for each threat level: 8 – critically endangered, 6 – threatened, 4 – vulnerable, 2 – data deficient and near threatened, and 0 – least concern;

We then calculated the Spearman rank correlation coefficient between each alternative scoring and the one used in our analysis in order to assess how the alternative scoring systems change the ranking of the grid cells based on the hotspots aggregated index (AI).

| Alternative scoring | ρ values  (relative to the scoring used for the hotspots prioritization) |
| --- | --- |
| 1 | 0.812*** |
| 2 | 0.981*** |
| 3 | 0.999*** |
| 4 | 0.999*** |

***p<0.0005
