# Supplementary material for: Conservation Planning for Biodiversity and Wilderness: A Real-World Example
Source: Environ Manage. 2015 Apr 3;55(5):1168–80. doi: 10.1007/s00267-015-0453-9 (PMC4392121; doi:10.1007/s00267-015-0453-9)
Supplement: Supplementary file 2 — Supplementary material 2 (DOCX 113 kb) [file 267_2015_453_MOESM2_ESM.docx]

Supplementary material 2

**Fig. S1.** Cumulative rare species representativeness plotted against the percentage of prioritized PNPG area according to the species-based and ecosystem-based approaches.

**Fig. S2.** Cumulative vulnerable species representativeness plotted against the percentage of prioritized PNPG area according to the species-based and ecosystem-based approaches.
